# Highlighting Free-Recovery and Work-Generating Shape Memory Effects at 80r-PET Thermoformed Cups

**DOI:** 10.3390/polym16243598

**Published:** 2024-12-23

**Authors:** Ștefan-Dumitru Sava, Bogdan Pricop, Mihai Popa, Nicoleta-Monica Lohan, Elena Matcovschi, Nicanor Cimpoeșu, Radu-Ioachim Comăneci, Leandru-Gheorghe Bujoreanu

**Affiliations:** Faculty of Materials Science and Engineering, “Gheorghe Asachi” Technical University of Iași, Blvd. Dimitrie Mangeron 71A, 700050 Iasi, Romania; stefan-dumitru.sava@student.tuiasi.ro (Ș.-D.S.); bogdan.pricop@academic.tuiasi.ro (B.P.); mihai.popa@academic.tuiasi.ro (M.P.); nicoleta-monica.lohan@academic.tuiasi.ro (N.-M.L.); elena.matcovschi@academic.tuiasi.ro (E.M.); nicanor.cimpoesu@academic.tuiasi.ro (N.C.); radu-ioachim.comaneci@academic.tuiasi.ro (R.-I.C.)

**Keywords:** shape memory polymer, shape memory effect, glass transition, storage modulus, tensile testing, work generation

## Abstract

The paper starts by describing the manufacturing process of cups thermoformed from extruded foils of 80% recycled PET (80r-PET), which comprises heating, hot deep drawing and cooling. The 80r-PET foils were heated up to 120 °C, at heating rates of the order of hundreds °C/min, and deep drawn with multiple punchers, having a depth-to-width ratio exceeding 1:1. After puncher-assisted deformation, the cups were air blown away from the punchers, thus being “frozen” in the deformed state. Due to the high cooling rate, most of the polymer’s structure reached a rigid, glassy state, the internal stresses that tended to recover the flat undeformed state were blocked and the polymer remained in a temporary cup form. When heating was applied, glass transition occurred, and the polymer reached a rubbery state and softened. This softening process released the blocked internal stresses and the polymer tended to recover its flat permanent shape. This relative volume contraction quantitatively describes the shape memory effect (SME) which can be obtained either with free recovery (FR-SME) or with work generation (WG-SME) when the cups lifted their bottoms with different loads placed inside them. The paper discusses the results obtained by differential scanning calorimetry (DSC), dynamic mechanical analysis (DMA), room-temperature tensile failure tests (TENS) and scanning electron microscopy (SEM). The DSC charts emphasized a glass transition, responsible for SME occurrence. The DMA thermograms and the TENS curves revealed that there are slight differences between the storage modulus and the tensile strains of the specimens cut on longitudinal, transversal, or 45° to the film rolling direction. The SEM micrographs enabled to observe structural differences between the specimens cut parallelly and transversally to the film’s rolling direction. The thermoformed cups were heated on a special experimental setup, which enabled the determination of FR-SME and WG-SME after applying different maximum temperatures and loads placed into the cups, respectively.

## 1. Introduction

The recovery of a permanent/high temperature-induced shape, by applying an external stimulus (such as heat, radiation, pH-change, or mechanical unloading) to a polymeric sample which was in a temporary/ low temperature-induced shape, is generally termed shape memory effect (SME) [1]. The SME-undergoing material systems, comprising metallic alloys, ceramics, polymers, composites and hybrids, are called shape memory materials (SMM) [2]. Among SMM, one of the most developed categories are shape memory polymers (SMP) [3].

In SMPs, there are several mechanisms that govern SME occurrence [4], such as the glass transition caused by heat, the photochromic reaction caused by irradiation, the magnetic domains rearranging caused by magnetic field, the pH-change caused by chemical activation, etc. [5].

Glass transition consists in the reversible transformation of SMP structure, from a rigid, glassy state to a soft, rubbery state during heating and vice versa during cooling [6]. The formation of the rigid, glassy state requires the application of rather high cooling rates, which are commonly used during various thermoforming processes [7].

In general, thermoforming is a continuous process consisting of five stages:

- heating the end of a thermoplastic foil roll beyond its glass transition softening temperature;
- deforming it, inside a metallic die, to the final shape, by vacuum-forming, pressure-forming, or mechanical forming [8];
- rapidly cooling it, between 40 °C to 60 °C, to ensure the formation of a solid, glassy state;
- cutting the final shape of the cup by trimming;
- ejecting the final product out of the die [9].

**Figure 1:**
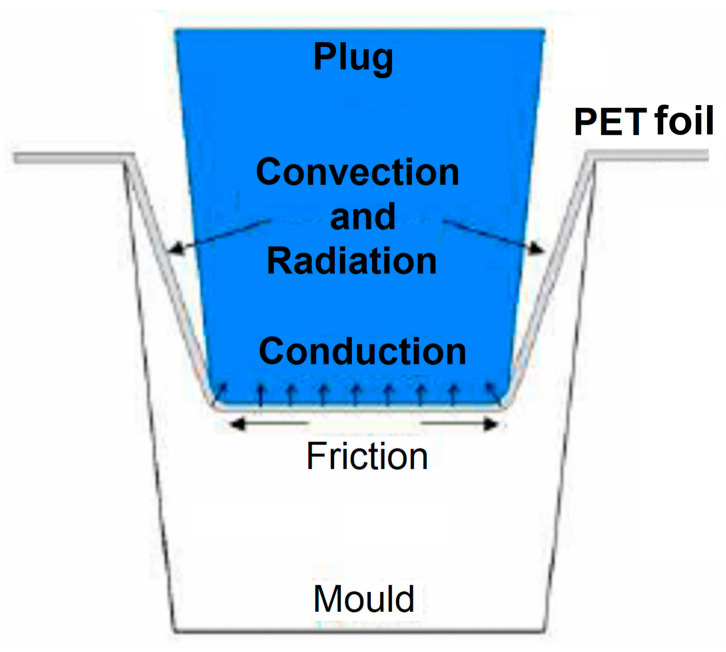
Schematization of heat transfer during deep drawing [10].

A schematization of the heat transfer mechanisms, during the general thermoforming process, is illustrated in Figure 1.

The following sequence of heat transfer occurs:

- by radiation, on both sides of the polyethylene terephthalate (PET) film, during initial foil heating;
- by conduction and convection, during deep drawing, in two stages:
- conduction is caused by the foil contact firstly with the puncher and then with the die, which is internally water-cooled;
- convection, due to rapid foil cooling caused by high-pressure air blowing (about 8 bar), after the die closure [10].

**Figure 2:**
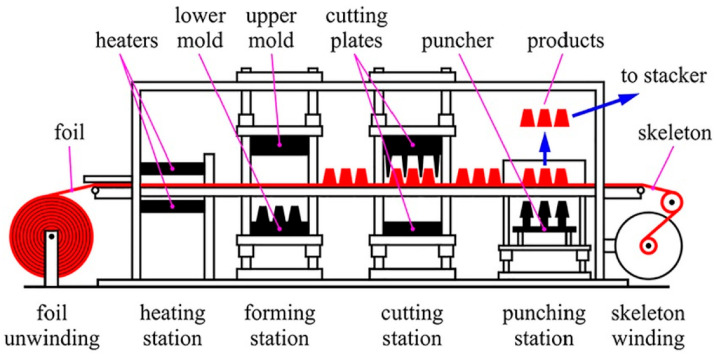
Schematic illustration of the thermoforming process [10].

Figure 2 illustrates a complete thermoforming line for cup production.

For the sake of a better comprehension, the four main steps of the thermophorming process have been separately illustrated. The die is composed of a lower part with punchers and a higher part with cavities. The four steps are:

- heating, when the foil is unwounded from the left side roll and passes between two heaters, being drawn by two lateral driving chains and reaches temperatures up to 120 °C in a matter of time up to 20 s;
- forming, during which the PET foil has a temporary stop, the die’s parts close and several punchers perform the air-assisted deep drawing process;
- cutting, while the foil is still stopped the die acts as a cutting station and the cups’ edges are trimmed and (iii) punching stations, respectively.
- air-ejection occurs after die opening, when high-pressure air is blown and the thermoformed cups are ejected into a stacker.

Finally, the foil skeleton is wound on the right-side roll. This continuous process is particularly efficient for the high-volume production of items, such as disposable cups [11].

Considering that the disposable cups are rapidly cooled, when ejected from the punchers by high-pressure air blowing, it can be expected that they will experience a certain amount of SME when heated, trying to recover their initial flat permanent shape.

Our previous results, obtained on filaments and 3D-printed parts from recycled polyethylene terephthalate (r-PET) and polyethylene terephthalate glycol (r-PETG), reported the development of SME, during the heating of deformed specimens. r-PET developed free-recovery (FR-SME) [12] and r-PETG experienced both FR-SME and work-generating SME (WG-SME) [13]. Based on these premises, the present article aims to investigate the capacity of r-PET thermoformed cups to develop FR-SME and WG-SME.

## 2. Materials and Methods

The raw material was an extruded rigid foil of 80% recycled PET (80r-PET) with a 1.05 mm thickness, a 650 mm width and a 361 m length, produced by Veroniki Ecogrup SRL, (Conțești, Romania). Due to the presence of 80% recycled PET, the foil’s color was translucent. The foil was used to produce disposable 350 mL cups, by means of a plastic cup thermoforming machine with ten punchers, type TQC-750, made by Litai Machinery Co., Ltd. (Wengzhou, China). Before deep drawing the foil was pre-heated between 90 and 120 °C, in less than 20 s, with a heating rate of several hundred °C/min. The thermoformed cups remained translucent but lost a part of their transparency at the end of the thermoforming process.

In order to characterize the 80r-PET foil’s thermal and mechanical properties, experiments were performed by differential scanning calorimetry (DSC), dynamic mechanical analysis (DMA) and room temperature (RT) tensile testing (TENS). The microstructure of 80r-PET specimens, cut along three different directions, was analyzed by scanning electron microscopy (SEM).

For DSC analysis, 3-mm diameter samples were punched with a laboratory die. The experiments were performed on a NETZSCH DSC 200 F3 Maia device (Netzsch, Selb, Germany), calibrated with Bi, In, Sn, Zn and Hg standards. Aiming to reveal the presence of glass transition, the specimens were placed into Al crucibles and heated at 10 °C/min, up to 300 °C, under an Ar protective atmosphere. Considering that glass transition occurs on a temperature range that is shifted to lower values by the decrease of heating rate [6], to avoid the occurrence of any artifact, a heating rate of 10 °C/min was used, which is typically recommended for DCS experiments [7]. The onset and end temperatures of glass transition were determined by the tangent method, being automatically calculated by the PROTEUS software (v.6.1, Selb, Germany): T_onset_: intersection of the left baseline tangent with the tangent at the inflection points; T_end_: intersection of the right baseline tangent with the tangent at the inflection point.

For DMA and TENS tests, parallelepipedal and “dog bone” specimens (with type 1BA geometry, according to EN-ISO 527-2 standard) were cut, respectively. Parallelepipedal specimens had 1.05 × 4 × 50 mm while tensile specimens had a gauge length of 25 mm [13]. The cutting of both DMA and TENS specimens was performed by means of a Gemini Andura Laser Cutter (Iasi, Romania), by choosing the specimen’s axis parallel to three orientations: (i) longitudinal (machine direction, MD), (ii) transversal direction (TD) and (iii) 45° regarding foil’s extrusion direction which coincides with the displacement direction during the thermoforming process.

DMA experiments were performed with a NETZSCH DMA 242 Artemis device equipped with a dual cantilever specimen holder. They were meant to reveal glass transition by storage modulus softening during heating, with a heating-cooling rate of 5 °C/min, while the specimen was dynamically bent with 1 Hz-frequency of and 100 μm-amplitude.

RT-TENS tests were performed up to failure on an INSTRON 3382 tensile machine (Norwood, MA, USA) and a cross-head speed of 1 mm/min. All the features of the machine can be found in our previous study [13].

SEM micrographs were recorded with a VEGA II LSH 132 TESCAN device (TESCAN, Brno—Kohoutovice, Czech Republic) on the cross section of the specimens that were broken by tensile tests, after depositing a 10 nm-thick gold layer, by means of a LUXOR Au/Pt Coater (APTCO, Berlin, Germany).

In order to test the shape memory behavior of the 350 mL thermoformed cups (weighing 8.3135 g), an experimental setup has been designed as an assembly comprising a 3D-printed skeleton that supports the cups and the heater. All of them were designed and 3D printed from polylactic acid (PLA) using a Prusa I3 printer (Prusa Research a.s., Prague, Czech Republic). A detailed description of the experimental device can be found in a previous paper where WG-SME was monitored during the lifting of various solid weights, by heating of the thermoformed cups [14].

For these particular experiments, the evolution of both FR-SME and WG-SME was investigated. The former consisted of air-heating within a furnace with controlled temperature. For the latter, the setup was adapted by changing the cups’ heating method from air blowing to a resistored water heating. Water’s temperature was accurately controlled using a solid state relay connected to a proportional-integral-derivative (PID) controller model AI-208 (Yudian Automation Technology, Hong Kong). The cups were filled with different amounts of water and heated up to five different temperatures (65 °C, 73 °C, 81 °C, 89 °C and 97 °C), by means of a resistive boiler. Knowing the initial volume, V_0_, of the water poured into the cup, the final volume, V_f_, was measured at the end of heating. The amount of shape recovery ratio (SME) is calculated with:

SME (%) = (V_0_ − V_f_)/V_0_ × 100 (1)

This formula considers the calculation principle of the shape recovery ratio of SMPs by dividing the difference between the maximum and measured deformation to the maximum one [15]. Five different water values of V_0_, were poured into different cups: 140 mL, 160 mL, 180 mL, 200 mL and 220 mL. These amounts were further considered as equivalent masses of water (in gf) that were lifted by each different glass by WG-SME developed during heating.

## 3. Results and Discussion

**Figure 3:**
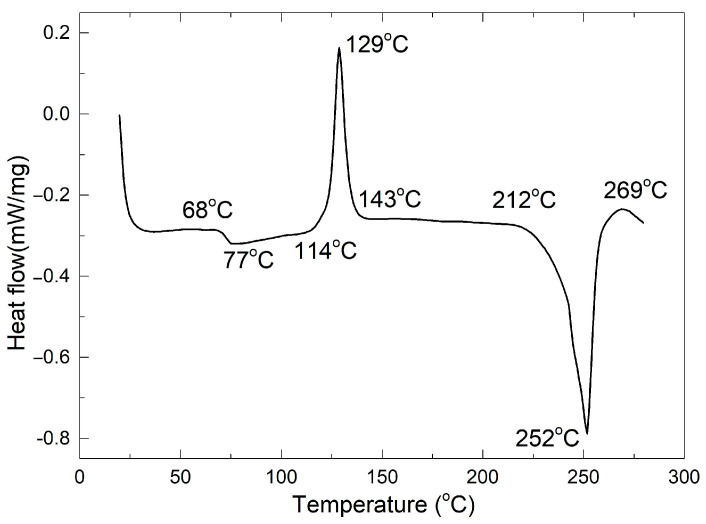
Typical DSC thermogram recorded during the heating of an 80R-PET fragment.

Figure 3 shows a typical DSC chart recorded during the heating of a punched fragment of 80r-PET rigid foil.

In good accordance with our previous observations [12], three transformations can be identified: (i) glass transition, between 68 and 77 °C; (ii) recrystallization, between 114 and 143 °C and (iii) melting, between 212 and 269 °C. These results confirm the presence of a glass transition that represents the prerequisite of SME occurrence.

Considering the maximum heating temperature of 120 °C of the foil, one could argue that, during thermoforming, the recrystallization process would have reached almost 50% at this stage, before cups deep drawing. However, due to the high heating rate and foil’s thermal inertia we can assume that the recrystallization range was not reached during heating.

**Figure 4:**
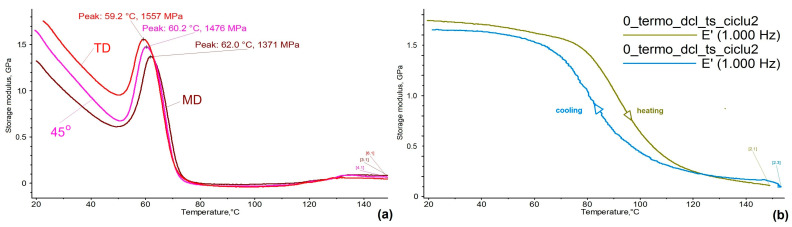
Representative DMA diagrams recorded during (**a**) the heating of specimens cut along three orientations about the foil’s extrusion direction; (**b**) the second heating-cooling cycle applied to the specimen cut along the foil’s extrusion direction.

DMA tests were performed on specimens cut along three different directions compared to the foil’s length. The main results, recorded during heating up to 150 °C (to avoid melting) are summarized in Figure 4a.

Figure 4a presents three diagrams of storage modulus variation during heating. As previously argued, the specimens were firstly destabilized during heating, which caused a sharp increase, up to about 1.5 GPa, and then the modulus decreased close to zero, which is a typical behavior for the glass transition [11]. The final modulus increase, located around 129 °C can be associated with recrystallization [16].

The evolutions of the three diagrams, from Figure 4a, suggest that a slight storage modulus stiffening occurred when changing the specimens’ cutting angle, from 0 (machine direction, MD) to 45° and finally to 90° (transverse direction, TD) regarding the foil’s extrusion direction. This minor anisotropy observed during dynamic bending could be caused by the presence of a small fraction of the crystalline triclinic phase, reported by Poluektov et al. [17], which could also be present in 80r-PET, considering that the molecular network has been in a tensile state in MD and a compressive state in TD [18].

On the other hand, Figure 4b displays a reversible variation of the storage modulus with temperature, in the second heating-cooling cycle, for the specimen cut in MD, where oriented molecules are mechanically less constrained than the ones oriented in TD [18]. As demonstrated by Yamanaka et al., non-oriented PET films experienced higher formability than the films that retained a faction of crystalline structure [19]. The reversible character of storage modulus variation suggests that the formation of the solid, glassy state occurred during cooling, since the modulus increased from approx. 150 MPa to about 1.7 GPa, at a cooling rate of 5 °C/min.

Further characterization of the mechanical behavior of 80r-PET was performed by static tensile tests. In each case, three failure tests were achieved with each of the three differently oriented specimens, 0° (MD), 45° and finally to 90° (TD) regarding the foil’s extrusion direction. The three tests were performed knowing that the tensile stress-strain curves of PET typically reveal a certain degree of scattering [20].

**Figure 5:**
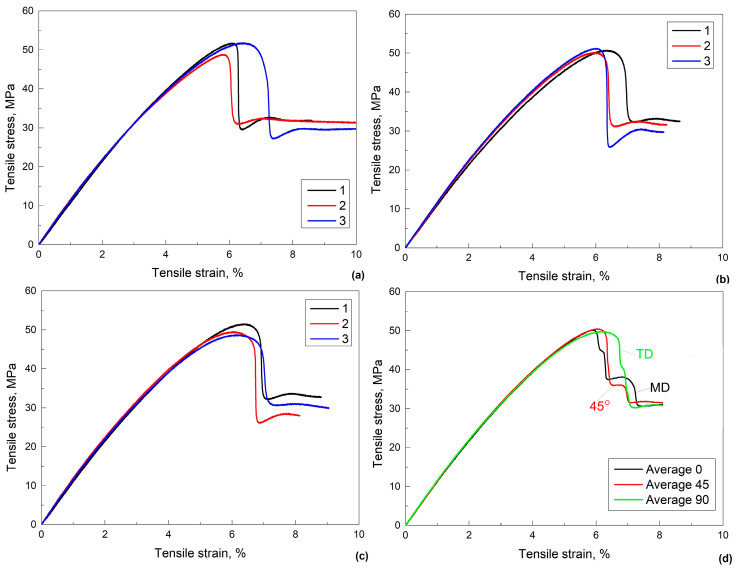
Static tensile failure curves of differently oriented specimens, regarding the foil’s extrusion direction: (**a**) 0° (MD); (**b**) 45°; (**c**) 90° (TD); (**d**) overlapping the three average failure curves.

The results of the tensile tests are summarized in Figure 5a–c. Figure 5d displays the average stress-strain curves of the above three orientations.

The stress variations with strain, of the three tensile failure curves recorded on specimens with the same orientation in Figure 5a–c, display a sharp stress decrease which is typically ascribed to necking [21].

Similar tensile failure curves, with sharp stress decrease, were reported for 3D printed specimens from r-PET filaments [22] and polyethylene terephthalate glycol (PET-G) [23], being typical for tough polymers with yield point as designated by standard [24].

According to the standard, Young modulus is calculated with the formula:

E = (σ_1_ − σ_2_)/(ε_1_ − ε_2_) (2)

where ε_1_ = 0.0005 and ε_2_ = 0.0025 while σ_1_ and σ_2_ are the stress values corresponding to these two strains.

Table 1 summarizes the parameters of strength and plasticity determined from the tensile stress-strain curves in Figure 5a–c.

The data from Table 1 confirm a certain scattering degree of the tensile stress-strain curves of PET. It is noticeable that, after the sharp stress decrease ascribed to necking, all of the nine curves displayed in Figure 5a–c, experienced an increase of stress, with as much as 50 MPa, during tensile strain increase with up to 1%.

The overlapping of the three average stress-strain curves, shown in Figure 5d, demonstrates that until the occurrence of the necking/yield point, the specimens behave almost identically, regardless of the cutting direction, compared to the foil’s extrusion direction. Nevertheless, after exceeding the necking/ yield point, the ultimate tensile strain increased in the succession 0° (MD) → 45° → 90° (TD), which agrees with the results reported by Meza de Luna and Shaikh [25].

**Figure 6:**
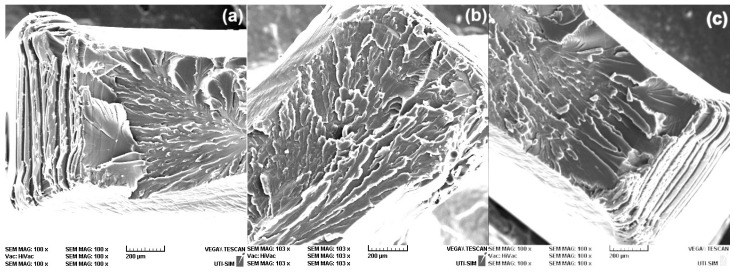
Typical low magnification SEM fractographs of the cross sections of differently oriented 80r-PET specimens, regarding the foil’s extrusion direction that failed according to Figure 5: (**a**) 0° (MD); (**b**) 45°; (**c**) 90° (TD).

Aiming to correlate the tensile behavior illustrated in Figure 5 with the structure of 80r-PET specimens, cut along the three different directions, a fractographic study was performed by SEM observations, on the tensile broken cross-section. The first characteristic micrographs, meant to explain necking occurrence, are illustrated in Figure 6.

At low magnification, no obvious differences are noticeable in the structure of the three specimens. However, the parallel stripes visible at the edges of the specimens from Figure 6a,c indicate the successive positions of the specimen’s surface during the necking, caused by tensile plastic deformation. In this way, the cross-section was suddenly reduced causing the sharp stress decrease observed in Figure 5.

**Figure 7:**
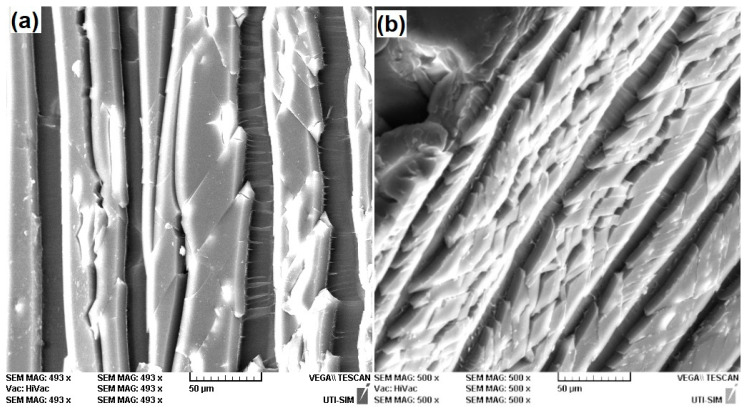
Typical SEM fractographs illustrating the presence of crazing (white) and delamination (dark) bands on the cross sections of differently oriented 80r-PET specimens: (**a**) 0° (MD); (**b**) 90° (TD).

For a better observation of the structural particularities of the specimens cut along MD and TD, higher-magnification SEM micrographs are illustrated in Figure 7.

The relatively higher plasticity of the TD specimen, as compared to the MD one, can be related to the increase in the number of consecutive failure layers [26], noticeable in Figure 7b, compared to Figure 7a.

Considering both fractograms, it can be assumed that the main failure mode was “crazing”, as illustrated by the white crack-like sharply localized bands of plastically deformed material [27]. In addition, both fractograms reveal dark bands that seem to be located below the surface of the crazing bands. Knowing that one of the particularities of tensile failure tests at polymers is the tearing of many fibers out of the matrix [28] and that the tensile failure curves from Figure 5 displayed a large amount of plastic deformation caused by necking, the dark bands could be caused by delamination [29]. The intricate relationship between the two deformation modes, crazing and delamination deserves deeper consideration and will be the subject of a next study.

The first type of experiment was meant to reveal the shape memory behavior of the 350 mL thermoformed cups. The cups lost a great part of their transparency due to the high amount of rapid high temperature deformation experienced by the foil, which reduced its thickness from 1.05 mm to 0.2 mm and to the color changing capacity of PET [30].

**Figure 8:**
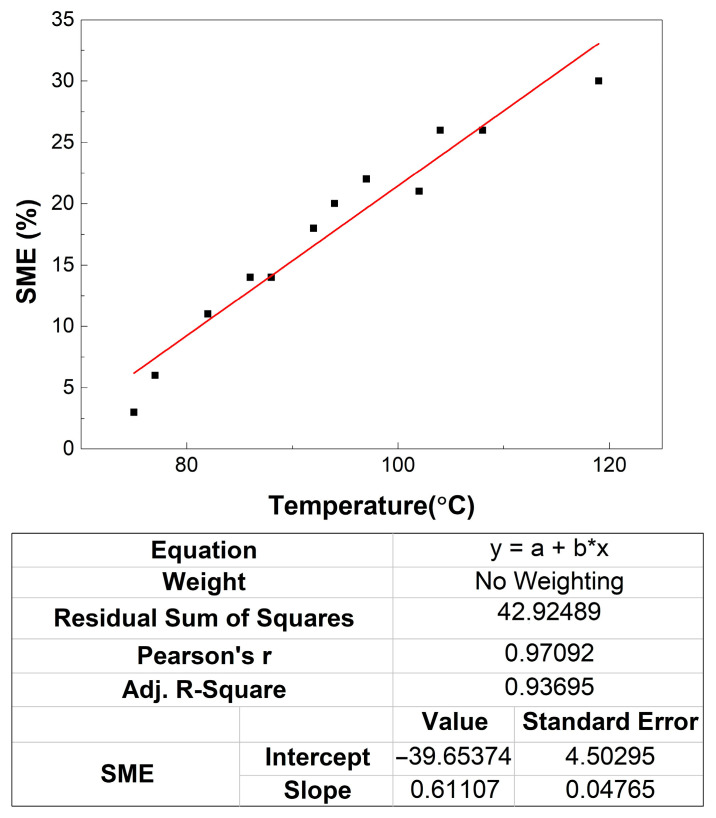
Variation with temperature of free recovery shape memory effect of 350 mL 80r-PET thermoformed cups. Dark squares are the experimental values and the red solid line is the linear fitting.

The experiments involved heating the cups in a furnace with a controlled temperature, measuring the final capacity, V_f_, after shape memory shrinkage and calculating FR-SME with Equation (1). The results are shown in Figure 8.

The linear fitting gives the relationship:

SME = 0.61107 T − 39.65374 (%), for T > T_g_ and SME for T < T_g_ (3)

where T is the temperature in °C.

Obviously, the “intercept” from Figure 8 has no physical interpretation, because there can be no SME is T = 0 °C. Aiming to verify the validity of the relationship (3), four new experiments were performed and the results are summarized in Table 2.

In Table 2 the following notations were used: SME^ex^—experimental values of FR-SME, SME^th^—theoretical values of FR-SME determined with Equation (3) and the error was calculated as (SME^ex^ − SME^th^)/ SME^ex^ × 100. It is noticeable that all error values are below 5%.

**Figure 9:**
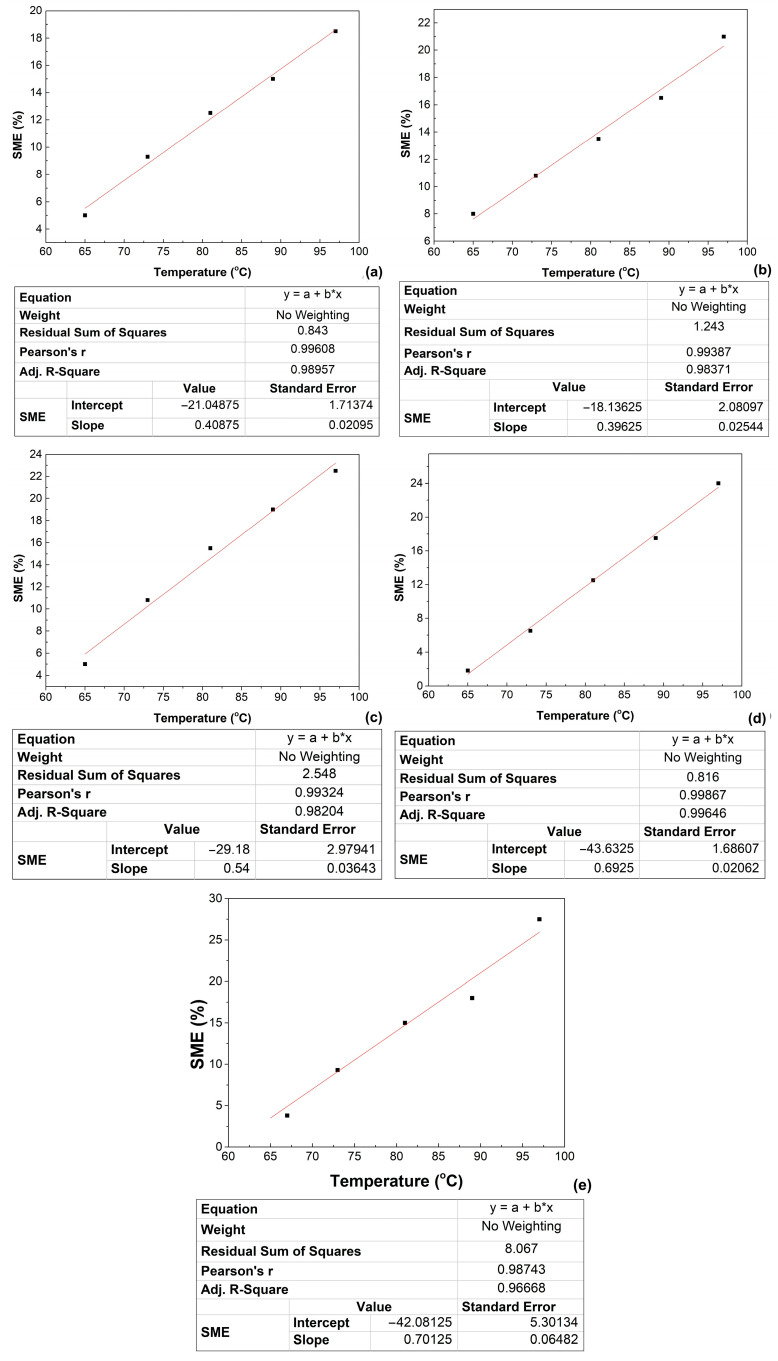
Variation with temperature of work generating shape memory effect of 350 mL 80r-PET thermoformed cups, filled with different amounts of water: (**a**) 140 g, (**b**) 160 g, (**c**) 180 g, (**d**) 200 g, (**e**) 220 g. Dark squares are the experimental values and the red solid line is the linear fitting.

The second type of experiment aimed to reveal the presence and evolution of WG-SME in the same 350 mL thermoformed cups. Actually, twenty five cups were filled with five different amounts of water (140, 160, 180, 200 and 220 g) and were heated up to five different accurately controlled temperatures (65 °C, 73 °C, 81 °C, 89 °C and 97 °C). The results are summarized in Figure 9.

The slopes of the linear variations of WG-SME with the temperature represent the rate of shape recovery percentage with temperature. Their values vary between 0.39625 and 0.70125%/°C.

**Figure 10:**
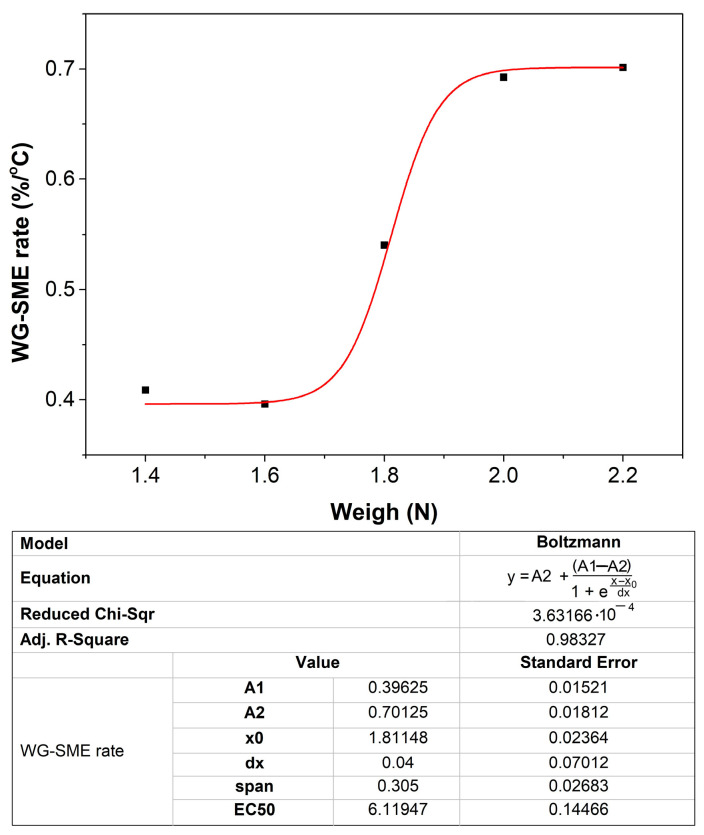
Variation, with the weight of filled water, of the work-generating shape memory percentage rate with temperature of 350 mL 80r-PET thermoformed cups. Black squares are experimental values and the solid line is the Boltzmann fitting function.

The variation of WG-SME rate with temperature vs. weight of the water mass filled into the thermoformed cups is illustrated in Figure 10.

As noticeable from Figure 10, the variation of WG-SME rate with temperature vs. the water weight was fitted with a Boltzmann-type function:

(4) $$y=A2+(A1-A2)/(1+e^{\frac{x-x_{0}}{dx}})$$

where y = WG-SME rate with temperature and x = weight of filled water.

At this point, it is worth mentioning that similar Boltzmann-type functions were also obtained, by some of the present authors, at the variations of free end’s displacement with temperature caused by FR-SME at r-PET [12] and r-PETG [13] filaments. This concordance requires further experiments to verify if other Boltzmann-type functions can be used to fit the variation of SME parameters with environmental conditions, for other polymeric specimens.

The saturation value of WG-SME rate with temperature and load weight is 0.7%/(°C).

The average vertical displacement of the bottom of the cups filled with the maximum amount of water (220 mL, the equivalent of approx. 2.2 N) was 1 cm. It follows that the work output has been W = 0.01 m × 2.2 N = 22 mJ. Considering the cup’s mass, 8.3135 g, it follows that the specific work output developed by a thermoformed cup has been W_spec_ = 0.022 J/0.0083135 kg ≈ 2.646 J/kg

## 4. Summary and Conclusions

An extruded rigid foil of 80r-PET with a 1.05 mm thickness was characterized by thermal, mechanical and structural analysis that enabled to draw the following conclusions:

- the thermal analysis was performed by DSC and temperature scan-DMA. DSC thermograms revealed the presence of a glass transition, between 68 and 77 °C which can substantiate the occurrence of SME. DMA diagrams emphasized a marked storage modulus increase during the first heating cycle and a reversible decrease during heating and increase during cooling in the second cycle;
- the tensile failure tests displayed a marked necking illustrated by sharp stress decrease and an increasing tendency of ultimate tensile strain, from machine direction to transversal direction;
- the structural analysis was performed by SEM that illustrated the successive positions of specimen’s surface that gradually decreased the cross section surface during necking and an obvious increase in the number of consecutive failure layers, when passing from machine to transversal direction.

The shape memory behavior of the 350 mL thermoformed cups was investigated by free-recovery (FR-SME) and work-generating (WG-SME) tests, which enabled the following conclusions:

- the variation of FR-SME with temperature was determined under the form of Equation (3) which was verified at four different temperatures and gave errors below 5%, thus enabling a correct determination of any SME value obtained during heating between 70 and 120 °C;
- the variation of WG-SME with temperature experienced rates of shape recovery percentage with temperature that varied between 0.39625 and 0.70125%/°C during the heating from 65 to 97 °C of thermoformed cups filled water amounts between 140 and 220 mL;
- the variation of the WG-SME rate with temperature and load weight was approximated with a Boltzman function, with standard errors below 0.15%, and reached a maximum value of 0.7%/(°C);
- the thermoformed cups developed both FR-SME and WG-SME, reaching maximum values of 30% and 25%, respectively;
- the maximum specific work output developed by a thermoformed cup was 2.646 J/kg.

Further investigations are necessary to elucidate the relationship between crazing and delamination failure modes at r-80PET and to verify the possible Botzmann-type fitting of the variation of SME parameters with environmental condition for other type of polymeric specimens.

## Figures and Tables

**Table 1:** Mechanical parameters determined from the tensile curves of Figure 5a–c.

Specimen		Maximum Load	Young Modulus	Yield Stress	Yield Strain	Tensile Stress	Tensile Strain
Orientation	No	N	MPa	MPa	mm/mm	MPa	%
0°	1	285.6	1065.01	44.88	0.05	51.74	6.1
	2	269.26	1211.59	32.72	0.03	46.75	5.26
	3	285.98	1159.71	35.98	0.04	49.8	5.69
45°	1	279.82	1062.59	41.48	0.04	50.69	6.37
	2	276.78	1095.84	42.88	0.04	50.14	5.96
	3	282.68	1175.99	38.6	0.04	51.21	6
90°	1	284.36	1058.19	43.45	0.05	51.51	6.36
	2	273.57	1212.8	34.54	0.03	49.56	6
	3	269.04	1185.38	34.53	0.03	48.74	6.16

**Table 2:** Results of checking the calculation relationship (3) of FR-SME.

Temperature	SME^ex^	SME^th^	Error
°C	%	%	%
81	10	9.48293	1.57
87	13.75	13.5094	1.75
96	20	19.009	4.96
112	30	28.7861	4.05

## Data Availability

The original contributions presented in this study are included in the article. Further inquiries can be directed to the corresponding author.
